# Improving biologic predictors of cycling endurance performance with near‐infrared spectroscopy derived measures of skeletal muscle respiration: E pluribus unum

**DOI:** 10.14814/phy2.14342

**Published:** 2020-01-20

**Authors:** Philip M. Batterson, Michael R. Norton, Sarah E. Hetz, Sachi Rohilla, Keston G. Lindsay, Andrew W. Subudhi, Robert A. Jacobs

**Affiliations:** ^1^ Department of Biology University of Colorado Colorado Springs Colorado Springs CO USA; ^2^ Department of Human Physiology and Nutrition University of Colorado Colorado Springs Colorado Springs CO USA; ^3^ Department of Health Sciences University of Colorado Colorado Springs Colorado Springs CO USA

**Keywords:** exercise, human performance, mitochondria, sport

## Abstract

The study aim was to compare the predictive validity of the often referenced traditional model of human endurance performance (i.e. oxygen consumption, VO_2_, or power at maximal effort, fatigue threshold values, and indices of exercise efficiency) versus measures of skeletal muscle oxidative potential in relation to endurance cycling performance. We hypothesized that skeletal muscle oxidative potential would more completely explain endurance performance than the traditional model, which has never been collectively verified with cycling. Accordingly, we obtained nine measures of VO_2_ or power at maximal efforts, 20 measures reflective of various fatigue threshold values, 14 indices of cycling efficiency, and near‐infrared spectroscopy‐derived measures reflecting in vivo skeletal muscle oxidative potential. Forward regression modeling identified variable combinations that best explained 25‐km time trial time‐to‐completion (TTC) across a group of trained male participants (*n* = 24). The time constant for skeletal muscle oxygen consumption recovery, a validated measure of maximal skeletal muscle respiration, explained 92.7% of TTC variance by itself (Adj *R* ^2^ = .927, *F* = 294.2, SEE = 71.2, *p* < .001). Alternatively, the best complete traditional model of performance, including VO_2max_ (L·min^−1^), %VO_2max_ determined by the ventilatory equivalents method, and cycling economy at 50 W, only explained 76.2% of TTC variance (Adj *R* ^2^ = .762, *F* = 25.6, SEE = 128.7, *p* < .001). These results confirm our hypothesis by demonstrating that maximal rates of skeletal muscle respiration more completely explain cycling endurance performance than even the best combination of traditional variables long postulated to predict human endurance performance.

## Graphical abstract

We compared the predictive validity of the often referenced traditional model of human endurance performance (i.e. oxygen consumption or power at maximal effort, fatigue threshold values, and indices of exercise efficiency) versus noninvasive near infrared spectroscopy‐derived measures of skeletal muscle oxidative potential in relation to endurance cycling performance across a group of healthy, trained participants (*n* = 24). This is the first study to show that when compared to 43 traditional variables long postulated to predict human endurance performance, measures reflecting maximal rates of skeletal muscle respiration seemingly stand alone as the best. E pluribus unum; out of many, one.

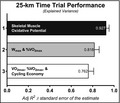

## 1 INTRODUCTION

Traditional exercise physiology dogma presents endurance performance aptitude as a biological formulate determined primarily through a combination of: (a) Measures reflecting one's maximal rate of whole‐body oxygen (O_2_) consumption (VO_2max_); (b) A valid fatigue threshold; and (c) An index of bioenergetic efficiency during exercise (Coyle, 1995; Gabriel & Zierath, 2017; Joyner & Coyle, 2008). More specifically, this established and accepted postulate suggests that one must maintain an external workload at a high percentage of their aerobic power (a product of VO_2max_ and fatigue threshold; “*performance VO_2_*”) that benefits from comparably higher metabolic (phosphorylative‐coupling) and mechanical (contraction coupling) efficiencies (Whipp & Wasserman, 1969) to maximize endurance performance. This premise has never been empirically verified collectively, to the best of our knowledge, and instead relies on implicit assumptions from the aggregation of literature examining human integrative physiology in relation to cycling endurance performance.

The physiologic mechanisms underlying aerobic power, fatigue thresholds, and exercise efficiency are not entirely independent from one another. The common biology shared across these variables relate to bioenergetic properties of skeletal muscle with emphasis on skeletal muscle mitochondria, as have been previously discussed (Gabriel & Zierath, 2017; Jacobs et al., 2011). Thus, a novel and more concise postulate advocates that skeletal muscle respiratory potential directs biological control of endurance performance potential. There is preliminary evidence in support of this theory (Jacobs et al., 2013, 2011). Accordingly, the intent of this study is to empirically scrutinize both postulates regarding the predictive physiology of endurance cycling performance in trained human participants.

The aims of the present study are to: (a) Verify that traditional variables commonly used to represent the capacity for exercise at maximal effort, a valid fatigue threshold, and an index of exercise efficiency, as is often discussed across the literature, do collectively account for a high percentage of cycling endurance performance (i.e. >75%); and (b) Demonstrate the close relationship between endurance performance and in vivo measures reflecting maximal skeletal muscle respiratory rates assessed with near‐infrared spectroscopy (NIRS), thereby expanding upon the traditional exercise physiology dogma of the physiologic limits of human performance. We hypothesize that the traditionally held formula used to describe endurance performance potential will aptly predict a high percent of endurance performance. However, we anticipate that measures indicative of maximal rates of skeletal muscle respiration will improve the physiological model explaining the potential of human endurance performance when cycling given the biologically based overlap of traditional variables and skeletal muscle respiratory capabilities.

## 2 MATERIALS and METHODS

### 2.1 General experimental design

#### 2.1.1 Participants

Study protocol and consent were approved by the Institutional Review Board at the University of Colorado Colorado Springs (UCCS; IRB 18‐122), and all experimental procedures conformed to the standards set by the latest revision of the Declaration of Helsinki. Study participants (male; *n* = 24) were informed of the experimental purpose, procedures, and risks before providing informed written consent to volunteer in the study. Participants were only considered eligible if they had regularly engaged in at least 3 days of endurance training per week for a minimum of 6 months prior to data collection. All participants were nonsmokers, determined capable of performing maximal exercise as per the recommendations of the American College of Sports Medicine (Riebe et al., 2015), free of any known cardiovascular, metabolic, and neurological diseases, and abstained from alcohol (24 hr), caffeine (12 hr), and exercise (48 hr) prior to data collection. Study participant characteristics are shown in Table 1.

**Table 1:** Participant characteristics and indices of cycling endurance performance. BMI, body mass index; TT^25^, 25‐km time trial; and TTC, time‐to‐completion

Variable	Mean ± *SD*	Relation to TTC
Sample size	*N* = 24	*(F‐statistic; p‐value)*
Time of day (hh:mm:ss)	13:20:03 ± 02:30:48	3.66; .0687
Temperature (°C)	21.7 ± 0.4	.01; .9163
Humidity (%)	21.9 ± 11.0	1.64; .2139
Age (years)	36.8 ± 11.6	.26; .6157
Height (cm)	180.4 ± 5.4	.19; .6695
Body mass (kg)	76.6 ± 9.8	.00; .9492
BMI	23.5 ± 2.6	.07; .7982
Time trial length (km)	25	
TTC (s)	2,680.9 ± 263.9	
Time per km (km·hr^−1^)	33.9 ± 3.3	
Average TT^25^ power (W)	214.2 ± 51.8	673.0; <.0001
Power‐to‐Weight ratio (W·kg^−1^)	2.84 ± 0.76	80.8; <.0001
Power‐to‐Weight ratio (W·kg^−0.32^)	53.6 ± 13.2	350.2; <.0001

#### 2.1.2 Study design

All experimental procedures were completed in the Human Performance & Bioenergetics Research Laboratory on the same day over ~4 hr at the Osborne Center for Science and Engineering, UCCS (~1,950 m elevation). All participants had ad libitum access to water throughout the study. The general study design, illustrated in Table 2, included an incremental ramp test to volitional fatigue (exercise test 1), a verification max test 10 min following the end of the initial incremental ramp test (exercise test 2), and then a 25‐km time trial (TT^25^), which commenced 60 min following the verification max test. Measures of power along with absolute (_abs_; l·min^−1^) and relative (_rel_; ml·kg^−1^·min^−1^) rates of whole‐body VO_2_ collected at maximal efforts from exercise tests 1 and 2 are represented by a superscripted number referencing the exercise test used to derive each measure. (^1^ or ^2^, respectively). All other variables do not include a superscripted number, as all other variables were derived from a single exercise test as explained subsequently and in Table 2.

**Table 2:**
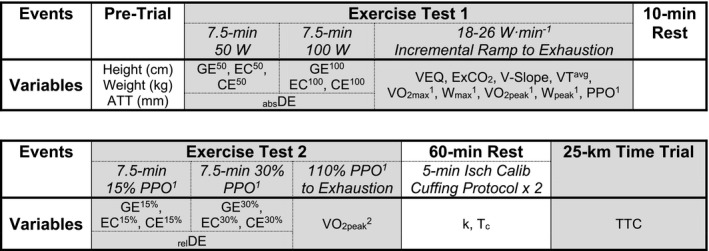
The experimental sequence of study design organizes measured variables with the events/test during which they were derived (read right to left; up to down)

Abbreviations: abs, absolute; ATT, adipose tissue thickness; CE, cycling economy; DE, delta efficiency; EC, exercise economy; ExCO_2_, excess CO_2_ method; GE, gross efficiency; Isch Calib, ischemic calibration; k, rate constant for the recovering rates of muscle oxygen consumption (mVO_2_); PPO, the power output at the point of exhaustion; rel, relative; T_c_, time constant for the recovery of mVO_2_; TT_25_, 25‐km time trial; TTC, time‐to‐completion; VEQ, ventilatory equivalents method; VO_2max_, maximal rate of whole‐body oxygen consumption; VO_2peak_, peak rate of whole‐body oxygen consumption; V‐Slope, modified V‐Slope method; VT^avg^, averaged ventilatory threshold (VT) values, including VEQ, ExCO_2_, and V‐Slope; W, watt; W_max_, averaged power output corresponding to VO_2max_; W_peak_, averaged power output corresponding to VO_2peak_.

#### 2.1.3 Exercise tests

Three exercise tests were performed on an electrically braked cycle ergometer (Velotron, Racermate Inc.), which allowed the use of the participant's own cycling shoes, pedal clips, and bike seat. Participants were fitted with a HR monitor (Polar T31, Polar Electro Inc., Bethpage) and continuous‐wave NIRS device (Oxymon MKIII, Artinis Medical Systems) for all exercise tests. Participants were also fitted with a head net and face mask combination (Hans Rudolph, Inc) with inspired and expired oxygen and carbon dioxide concentrations continuously measured and analyzed as breath‐by‐breath values (Ultima CPX, Medgraphics Diagnostics) to assess VO_2_ and rates of carbon dioxide production (VCO_2_) during exercise tests 1 and 2. The gas analyzers and flowmeters were calibrated before each study. Participants were instructed to refrain from interrupted breathing while ventilatory measures were collected.

Exercise test 1 consisted of a pre‐programmed standardized warm‐up and incremental ramp to volitional exhaustion (Velotron Coaching Software). This test was designed to: (a) Collect measures of exercise efficiency at the same absolute workloads during a standardized warm‐up; (b) Determine various measures of fatigue threshold during an incremental ramp protocol; and (3) Calculate VO_2max_^1^ with corresponding averaged measures of maximal power output (W_max_^1^), VO_2peak_^1^ with corresponding average measures of peak power output (W_peak_^1^), and peak power output (PPO^1^), which was the power output at the point of exhaustion (Table 2). A more complete description of these calculations is detailed below. The 15‐min standardized warm‐up consisted of cycling at two sequential 7.5 min workloads of 50‐and‐ 100 W at a set cadence of 80 rpm. Immediately following the 100 W stage, participants were free to pedal at a self‐selected cadence as the workload was increased by 18–26 W·min^−1^ (dependent upon age, BMI, and cycling experience) until achieving volitional exhaustion, which was defined as a drop in cycling cadence < 55 rpm. Participants were verbally encouraged to continue cycling to complete exhaustion once a consistent RER > 1.00 was identified. Following cessation of exercise test 1, participants rested on the cycle ergometer for 10 min as PPO^1^ was determined and subsequently used to program exercise test 2 (Velotron Coaching Software; RacerMate).

Exercise test 2 was designed to ensure valid measures of VO_2max_^1^ were collected in exercise test 1 (Poole & Jones, 2017) and to collect measures of exercise efficiency across the same relative constant workloads (Table 2). This test commenced after 10‐min of rest and/or light active recovery, as participants completed two sequential 7.5 min workloads set to 15% and 30% PPO^1^, respectively, again at a set cadence of 80 rpm. Immediately following the 30% PPO^1^ stage, workload increased to 110% PPO^1^ over a 60 s interval. Thereafter, participants continued to pedal at a self‐selected cadence at 110% PPO^1^ until volitional exhaustion (cadence < 55 rpm). Participants were again verbally encouraged to continue cycling to complete exhaustion throughout the final workload. Following exercise test 2, participants rested for one hour before completing a TT^25^.

Fifteen minutes prior to beginning the TT^25^, all participants were provided an isocaloric beverage with a standard carbohydrate load of 36 g (Gatorade, PepsiCo) that had to be consumed prior to beginning the time trial (*n* = 24) to normalize participant nutrition and provide standardized exogenously available substrate for the TT^25^. After resting for one hour following completion of exercise test 2, participants completed an all‐out TT^25^ (Racermate One Software, SRAM Inc.) to assess endurance cycling performance. They were instructed to complete the TT^25^ as quickly as possible using the same cycle ergometer individually fitted to each participant prior to exercise test 1, with the ability to freely adjust their power output, similar to shifting gears, at any given time throughout the entire test. Participants received restricted temporal feedback during the TT^25^, limited to verbal confirmation for every 5‐km completed for the initial 20 km, and then every 1‐km for the final 5 km. Our index of cycling endurance performance derived from this test was time‐to‐completion (TTC).

### 2.2 Methodology for collecting traditional physiologic variables relating to human endurance performance

#### 2.2.1 Determination of VO_2_ and power output at maximal effort

VO_2_ and cycling power were derived at maximal efforts using multiple methods to account for a wide array of concerns regarding measures derived at maximal exercise (Poole & Jones, 2017). Least squares linear regression was used to identify the highest but most stable (flattest slope) average VO_2_ across the greatest range of time to determine VO_2max_^1^ with corresponding measures of W_max_^1^ derived as the average power across that same range of time. Alternatively, VO_2peak_^1 & 2^ were determined as the highest 30 s average VO_2_ throughout each respective exercise test with W_peak_^1^ values reflecting average measures of power output across that same range of time. Given our experimental design:

$$1.10\times {\text{PPO}}^{1}=W_{\max^{2}}=W_{{\text{peak}}^{2}}={\text{PPO}}^{2}$$

As the verification test was programmed to ramp up to a constant power output equal to 110% of PPO^1^, all measures of power in the verification max test, exercise test 2, are inherently collinear with PPO^1^. Consequently, all measures of cycling power used for statistical analyses were limited to those derived from exercise test 1.

#### 2.2.2 Determination of fatigue thresholds

Identification of fatigue thresholds using methods to determine ventilatory threshold (VT) have been identified as superior (Amann, Subudhi, & Foster, 2006) or equivalent (Aunola et al., 1988; Cerezuela‐Espejo, Courel‐Ibáñez, Morán‐Navarro, Martínez‐Cava, & Pallarés, 2018; Pallarés et al., 2016) to measures and/or surrogates (Black, Durant, Jones, & Vanhatalo, 2014) of lactate threshold (LT). As such, three common methods to determine VT were evaluated, as previously explained (Gaskill et al., 2001). Briefly, the methods evaluated include the following:

1. *Ventilatory equivalent method (VEQ)*: The intensity of activity that causes the first rise in the ventilatory equivalent of oxygen (VE/VO_2_) without a concurrent rise in the ventilatory equivalent of carbon dioxide (VE/VCO_2_).
2. *Excess carbon dioxide method (ExCO_2_)*: The intensity of exercise that causes an increase from steady state to an excess production of CO_2_ and is calculated as ((VCO_2_^2^/VO_2_)−VCO_2_).
3. *Modified V‐slope method (V‐slope)*: The intensity of exercise that, in a plot of the VCO_2_ over the VO_2_, shows an increase in the slope from less than 1 to greater than 1. This method was modified from the original method (Beaver, Wasserman, & Whipp, 1986) that used breath‐by‐breath gas analysis to the use of 5‐s gas collection averages.

Corresponding fatigue threshold values of _abs_VO_2_, _rel_VO_2_, %VO_2max_, workload (W), and %W_max_ were determined using each method. All measures respective to each VT method (methods 1–3 explained above) were also averaged (VT_AVG_), because the average of these three methods is postulated to be more resistant to false VT determinations and better reflect one's VT (Gaskill et al., 2001).

#### 2.2.3 Determination of cycling efficiency

Four common methods to determine cycling efficiency were evaluated, as previously described (Lucía, Hoyos, Pérez, Santalla & Chicharro 2002; Moseley et al., 2004; Moseley & Jeukendrup, 2001). Bioenergetic efficiency of cycling was assessed by determining gross efficiency (GE), exercise economy (EC), and cycling economy (CE) at the same absolute (50‐ and‐ 100 W) and relative (15% and 30% PPO^1^) steady‐state workloads, as well as delta efficiency (DE) across the same absolute and relative steady‐state workloads. Calculations of GE, EC, CE, and DE were derived from measures of energy expenditure, VO_2_, and work rate. Energy expended (EE) was calculated from the measures of VCO_2_ and VO_2_ (Ultima, Medgraphics Diagnostics) and analyzed using the formula:

$$\begin{array}{cc} \text{Energy Expenditure}\left(J\cdot s^{-1}\right) & =\text{Watts}\left(W\right)=\left[\left(3.869\times {\text{VO}}_{2}\right)+\left(1.195\times {\text{VCO}}_{2}\right)\right]\\ & \;\times \;\left(4.186/60\right)\times 1000. \end{array}$$

GE was calculated as the mean of the most level portion over the latter portion of each steady‐state workload:

$$\text{GE}\left(\%\right)=\left(\text{Work Rate}\left(W\right)/\text{Energy Expended}\left(W\right)\right)\times 100\%$$

EC was calculated as the power output divided by the rate of O_2_ consumption using the most level portion of the latter portion of each steady‐state workload and expressed in kJ·L^−1^; CE was expressed in (W·L^−1^·min^−1^); and both absolute (_abs_) and relative (_rel_) measures of DE were calculated as change in work performed divided by the change in energy expenditure (Jacobs et al., 2011).

Metabolic steady‐state is achieved within 3–6 min of exercise at a constant workload, especially at relatively lower submaximal workloads (Gaesser & Brooks, 1975; Wasserman et al., 1973; Whipp & Wasserman, 1972). To ensure that steady‐state was reached during all four constant‐load stages (Table 2), each stage consisted of cadence‐controlled (metronome set to 80 rpm) fixed‐effort cycling for 7.5 min stages. To ensure that end‐stage averaged values reflected true steady‐state conditions, least squares linear regression was used to identify the most level portion (flattest slope) across the end of each constant‐load stage for exercise efficiencies derived during all submaximal stages of exercise tests 1 and 2.

### 2.3 Methodology for collecting maximal rates of skeletal muscle respiration via NIRS

#### 2.3.1 Ultrasound (US)

Thigh adipose tissue thickness (ATT) was measured using B‐mode ultrasound (VIVID e; GE HealthCare), as previously described (Müller et al., 2013). Briefly, randomized and counter‐balanced measurements were made in a supine position superficial to the left or right *m. vastus lateralis *~10 cm superior to the proximal edge of the patella. The US probe was placed, using minimal pressure, parallel to the direction of the *m. vastus lateralis* with 3–5 mm of US gel (Aquasonic Clear; Parker Laboratories, Inc., Fairfield) between the probe and skin. All measures of ATT were collected by the same research assistant with the US system set to ‘default’ except for frequency and time‐gain compensation. All scans were repeated until image resolution was sufficiently clear for the subcutaneous fat‐skeletal muscle interface to be identified. Measures of ATT, in millimeters, were taken to be the average of three points, determined via software calipers, across the image.

#### 2.3.2 NIRS device

Continuous‐wave NIRS (Oxymon MKIII, Artinis Medical Systems) optodes were placed on the skin over the *m. vastus lateralis*. As previously described (Ryan, Southern, Reynolds, & McCully, 2013), the probe was set to have two source‐detector separation distances (between 30 and 45 mm), with the smallest source‐detector distance set to approximately twice the ATT. The second source‐detector distance was set 1 cm greater than the first. NIRS data were collected at 10 Hz. All statistical analyses included only NIRS‐derived measures of deoxygenated hemoglobin and myoglobin (HHbMb). Continuous‐wave derived measures of HHbMb are less influenced by skin blood flow immediately below the sensors allowing for a valid estimate of local muscle fractional O_2_ extraction (Koga et al., 2015).

#### 2.3.3 Ischemic/hyperemic calibration

Immediately following exercise test 2, participants were seated on a reclinable chair (Ostrich Deluxe Chair, Detless Corp.) with their feet elevated approximately parallel to the floor, while an ischemic calibration procedure was performed. Blood pressure cuffs (Hokanson SC12D) were placed proximal to the NIRS optodes on the upper thigh of each leg and connected to a rapid‐inflation system (Hokanson E20). The blood pressure cuffs were inflated to ~300 mmHg for 5 min (or until the HHbMb signal plateaued). An ischemic/hyperemic occlusion protocol was used to normalize the HHbMb signals to the maximal physiological range of each participant. The highest value obtained represented zero oxygenation in the tissue under the NIRS probe. Upon release of the blood pressure cuff, a hyperemic response causes a re‐oxygenation overshoot in the NIRS signal, with the minimum HHbMb identified to represent 100% oxygenation.

#### 2.3.4 Leg occlusion cuffing protocol

Noninvasive determination of skeletal muscle O_2_ consumption (mVO_2_) recovery rates were determined using a protocol similar to several previously described (Ryan, Brizendine, & McCully, 2013; Ryan et al., 2014; Ryan, Southern, Reynolds, et al., 2013). Immediately following the ischemic/hyperemic calibration, while remaining seated in a recumbent position, participants performed near‐maximal to maximal voluntary isometric leg extension contractions for 2 × 10 s contraction/10 s relaxation, followed by one 20 s contraction (60 s in total), which led directly into the occlusion cuffing protocol. Increased measures of mVO_2_ between isometric contractions and electrically stimulated contractions have been shown to be similar and independent of exercise intensity (Ryan, Brizendine, et al., 2013). Immediately after completion of the 1 min isometric contraction protocol, the blood pressure cuffs were inflated to ~300 mmHg for a series of 5–15 s occlusions to measure mVO_2_ recovery kinetics. The cuffing protocol consisted of 15 cuffs as follows: cuffs 1–5 (5 s on/off), 6–10 (10 s on/off), 11–15 (15 s on/off). The isometric contractions and leg occlusion cuffing protocols were completed twice for duplicate measures and values determined for each protocol were averaged.

#### 2.3.5 Rates of skeletal muscle O_2_ consumption recovery

Rates of mVO_2_ recovery were calculated using simple linear regression across 3 s of each occlusion (30 data points × 15 occlusions). The post exercise repeated measurements of mVO_2_ were fit to a monoexponential curve according to the formula below:

$$y\left(t\right)=\left(\text{end}-\text{delta}\right)\times e^{k\;\times \;\;t}$$

where *y(t)* is the relative mVO_2_ during the arterial occlusion at time *t*, end is the mVO_2_ immediately following exercise, delta is the change in mVO_2_ from rest to end‐exercise, and *k* is the fitting rate constant. According to this first‐order metabolic model, the recovery kinetics follow a monoexponential function that is independent of exercise intensity (Crow & Kushmerick, 1982; Mahler, 1985; Meyer, 1988; Paganini et al., 1997; Ryan, Brizendine, et al., 2013; Walter et al., 1997). The rate constant k correlates with maximal rates of skeletal muscle respiration determined via high‐resolution respirometry (Ryan et al., 2014). The corresponding time constant (T_c_) for the recovery of mVO_2_ (1·k^−1^) correlates with PCr recovery kinetics via ^31^phosphorus‐magnetic resonance spectroscopy (Ryan, Brizendine, et al., 2013).

#### 2.3.6 Correction for blood volume

All NIRS data obtained during the ischemic calibration and cuffing occlusion protocol were corrected for blood volume changes as previously described (Ryan et al., 2012). Briefly, it is assumed that oxygenated hemoglobin and myoglobin (O_2_HbMb) and HHbMb change at a 1:1 ratio during arterial occlusions with the resulting signals representative of mVO_2_, and the calculation of a blood volume correction factor (β) takes these changes into account. To correct NIRS signals for changes in blood volume, first the following equation was employed to determine a correction factor for each data point obtained during occlusions:

$$\beta \left(t\right)=O_{2}\text{HbMb}\left(t\right)/\left(O_{2}\text{HbMb}\left(t\right)+\text{HHbMb}\left(t\right)\right).$$

Each data point was then corrected using its corresponding β in the equations shown below. This method for correcting each data point for its corresponding blood volume has been empirically validated (Ryan et al., 2012).

$$O_{2}{\text{HbMb}}_{c}=O_{2}\text{HbMb}-\left[\text{THbMB}\times (1-\beta )\right].$$

$${\text{HHbMb}}_{c}=\text{HHbMb}-\left[\text{THbMb}\times \beta \right].$$

In these equations, O_2_HbMb_c_ and HHbMb_c_ are the corrected O_2_HbMb and HHbMb signals respectively.

### 2.4 Statistical analyses

Laboratory‐based stationary time trial cycling tests do not seamlessly translate to real‐world performance, as cyclists with a larger body mass experience greater air resistance, primarily due to increased frontal area, when riding outside of laboratory conditions on flat terrain (Bassett, Kyle, Passfield, Broker, & Burke, 1999; Swain, 1994; Swain et al., 1987). Relative measures of VO_2_ consumption and all measures of power output were allometrically controlled for body mass‐surface area relationships using the mass exponents 0.32 to account for differences across cyclists and improve upon the real‐world application of these findings (Swain, 1994).

Comparisons between two parametric or nonparametric variable groups were evaluated using a paired *t*‐test or Mann–Whitney test respectively (GraphPad Prism 8.1.2, GraphPad Software). Comparisons across three or more groups were analyzed using a one‐way ANOVA or repeated measurements ANOVA with a Bonferroni correction to control for type I error across multiple comparisons. When appropriate, post hoc tests were conducted using Bonferroni multiple comparison test.

In total, we obtained nine variables at maximal exercise, 20 measures reflective of various fatigue threshold values, 14 indices of cycling efficiency, along with k and T_c_ for examination of variable modeling to best explain TTC variance. The most predictive variable(s) from each tier representing different ‘traditional’ variable categories (i.e. max exercise, fatigue threshold, & exercise efficiency values) were first identified with linear regression or forward regression analysis (SigmaPlot, Systat). For statistical modeling of cycling endurance performance, there were two objectives: (a) Substantiate that measures of VO_2_ or power at maximal efforts, our strongest fatigue threshold value in relation to TTC, and our best measure(s) of exercise efficiency, as often discussed across the literature, do collectively account for a high percent (>75%) of variation of cycling endurance performance, delineated by a TT^25^, across a group of active, well‐trained participants; and (b) Examine whether including in vivo rates of skeletal muscle respiration improve upon traditional regression models explaining endurance performance.

Best‐fit grouped variables identified through forward stepwise regression analyses were assessed with a correlation matrix (*R* ^2^ ≥ .80; Figure 2) and multiple linear regression (variance inflation factor, VIF ≥ 2.0) to further scrutinize variable collinearity. The variable with the stronger/est relation to TTC was retained for further regression model analyses if collinearity was identified, as other collinear variables were removed.

For all statistical evaluations, an α of *p* < .05 was considered significant and all data are reported as mean ± *SD* unless specified otherwise.

## 3 RESULTS

### 3.1 Experimental design validation and group comparison analyses

Participant characteristics and indices of TT^25^ cycling endurance performance are displayed in Table 1. Neither testing conditions (time of testing, temperature, and humidity) nor subject characteristics (age, height, weight, and BMI) significantly related to TTC (*p* > .05).

#### 3.1.1 Exercise tests

There were no differences in average cycling cadence across all four constant‐load submaximal 7.5 min stages (79.9 ± 1.4, 80.4 ± 0.8, 80.4 ± 0.8, and 80.5 ± 0.6) for 50 W, 100 W, 15% PPO^1^, and 30% PPO^1^ exercise intensities respectively; *p* > .05). The time to fatigue for incremental ramp protocols (18–26 W·min^−1^) in exercise test 1 were consistent across all participants, averaging 10 min 55 s ± 1 min 26 s.

### 3.2 Identification of best traditional physiologic variables relating to human endurance performance

#### 3.2.1 Determination of the best indices of cycling efficiency in relation to human endurance performance

Indices of exercise efficiency significantly related to TTC are shown in Figure 3a. All relative measures of GE, EC, and CE were related to TTC whereas only one absolute measure was significant, GE^50^. Measures of efficiency, such as GE, are known to be positively influence by workload (Gaesser & Brooks, 1975; Moseley & Jeukendrup, 2001). Indeed, relative measures of GE (Figure 3b), EC (Figure 3c), and CE (Figure 3d) were influenced by workload such that more ‘efficient’ measures correlated with higher workloads. Relative efforts during constant‐load cycling at 15% and 30% PPO^1^ are inherently colinear to PPO^1^ as per our study design and thus average power determined at maximal effort, W_max_, also strongly correlates with power at 15% and 30% (both, *R* ^2^ = .9295). Accordingly, these relative measures of exercise efficiency were removed from further grouped regression analyses as the weaker correlates. Instead, absolute indices of exercise efficiency that demonstrated a relationship with TT^25^ performance (*F* > 2.0) were retained for subsequent grouped forward regression analyses. These indices included GE^50^, EC^50^, CE^50^, GE^100^, and EC^100^ (Figure 4).

#### 3.2.2 Determination of best methodology to identify values at fatigue threshold in relation to human endurance performance

There were no significant main effects (all, *p* < .05) for methodology used to determine corresponding fatigue threshold values of _abs_VO_2_ (*F* = 3.68), _rel_VO_2_ (*F* = 3.74), %VO_2max_ (*F* = 3.30), W (*F* = 2.52), and %W_max_ (*F* = 2.09). Although, comparisons across methods to determine _abs_VO_2_ (*p* = .057), _rel_VO_2_ (*p* = .055), and %VO_2max_ (*p* = .072) did show trends of difference driven solely by disparities between VEQ and ExCO_2_ values (Table 3). Also, fatigue threshold powers did not differ from the average power throughout the TT^25^ (*F* = 2.453, *p* = .1112; Figure 2a).

**Table 3:** Fatigue threshold values across methodologies. All fatigue thresholds were derived from exercise test 1 and were assessed via repeated measurements ANOVA with a Bonferroni correction to control for type I error across multiple comparisons

Method	_abs_VO_2_	_rel_VO_2_	%VO_2max_	W·kg^−0.32^	%W_max_
*VEQ*	2.56 ± 0.49	640.8 ± 119.3	63.4 ± 8.1	49.3 ± 12.0	56.2 ± 9.0
*ExCO_2_*	2.72 ± 0.60	678.5 ± 147.4	66.9 ± 9.7	52.6 ± 13.9	59.8 ± 10.9
*V‐Slope*	2.73 ± 0.70	682.0 ± 173.1	67.2 ± 13.1	53.4 ± 16.9	60.6 ± 15.3
*VT_avg_*	2.67 ± 0.57	667.1 ± 140.5	65.8 ± 9.4	51.8 ± 13.7	58.9 ± 10.9

Note

Fatigue threshold variables consist of absolute (_abs_; l O_2_·min^−1^) and relative (_rel_; ml O_2_·min^−1^·kg^−0.32^) measures of oxygen consumption (VO_2_) or power output (W·kg^−0.32^), as well as % of maximal VO_2_ (%VO_2max_) or power (%W_max_) determined through different methods: ventilatory equivalent method (VEQ); excess carbon dioxide method (ExCO_2_); modified V‐slope method (V‐slope); and the average of VEQ, ExCO_2_, and V‐Slope methods (VT^avg^). Data reported as mean ± *SD*.

**Figure 1:**
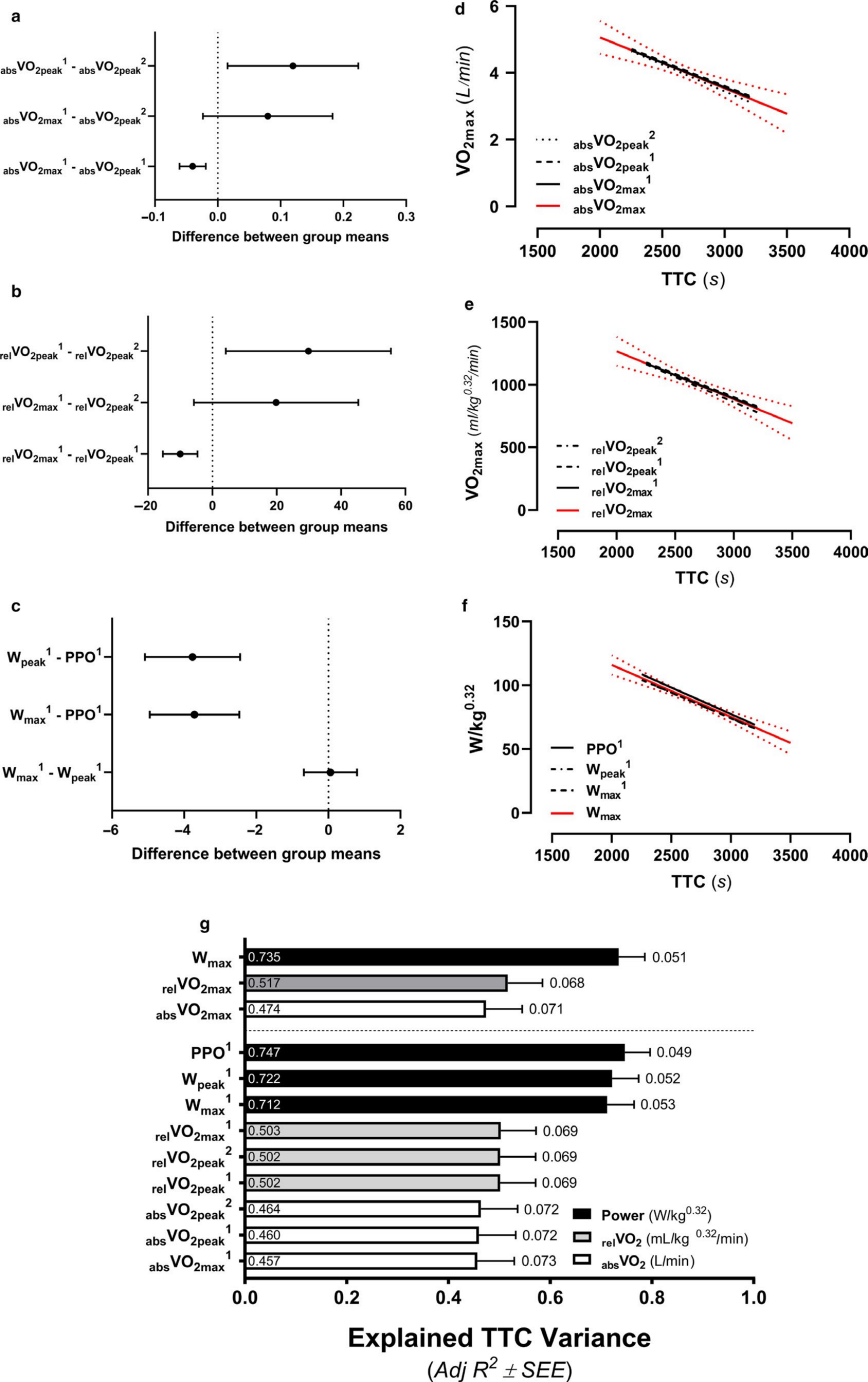
Determination of the best measures for rates of whole‐body oxygen consumption (VO_2_) and power at maximal efforts in relation to endurance performance. The median difference and 95% confidence interval between methods for determining absolute (_abs_; L·min^−1^) VO_2_ (a), relative (_rel_; ml·min^−1^·kg^−0.32^) VO_2_ (b), and power (W·kg^−0.32^; c) at maximal efforts. The relationship between _abs_VO_2_ (d), _rel_VO_2_ (e), and power (f) in relation to 25‐km time trial time‐to‐completion (TTC). The red lines represent the respective averages of corresponding measures collected during exercise tests 1 (^1^) and 2 (^2^) and the dotted red line represents 95% confidence interval. Linear regression relationships between traditional variables collected at maximal efforts in relation to TTC (g). The labels within bars reports individual Adj R^2^ values representing explained variance and the labels outside of the error bars represents the standard error of the estimate (SEE) as a percent of the averaged group TTC (2,680.9 s). The highest but most stable (flattest slope) average VO_2_ across the greatest range of time was used to determine VO_2max_^1^ with corresponding measures of W_max_^1^ derived as the average power across that same range of time. Alternatively, VO_2peak_^1 & 2^ were determined as the highest 30 s average VO_2_ throughout each respective exercise test with W_peak_^1^ values reflecting average measures of power output across that same range of time. Peak power output (PPO^1^) was determined as the power output at the point of exhaustion for exercise test 1

**Figure 2:**
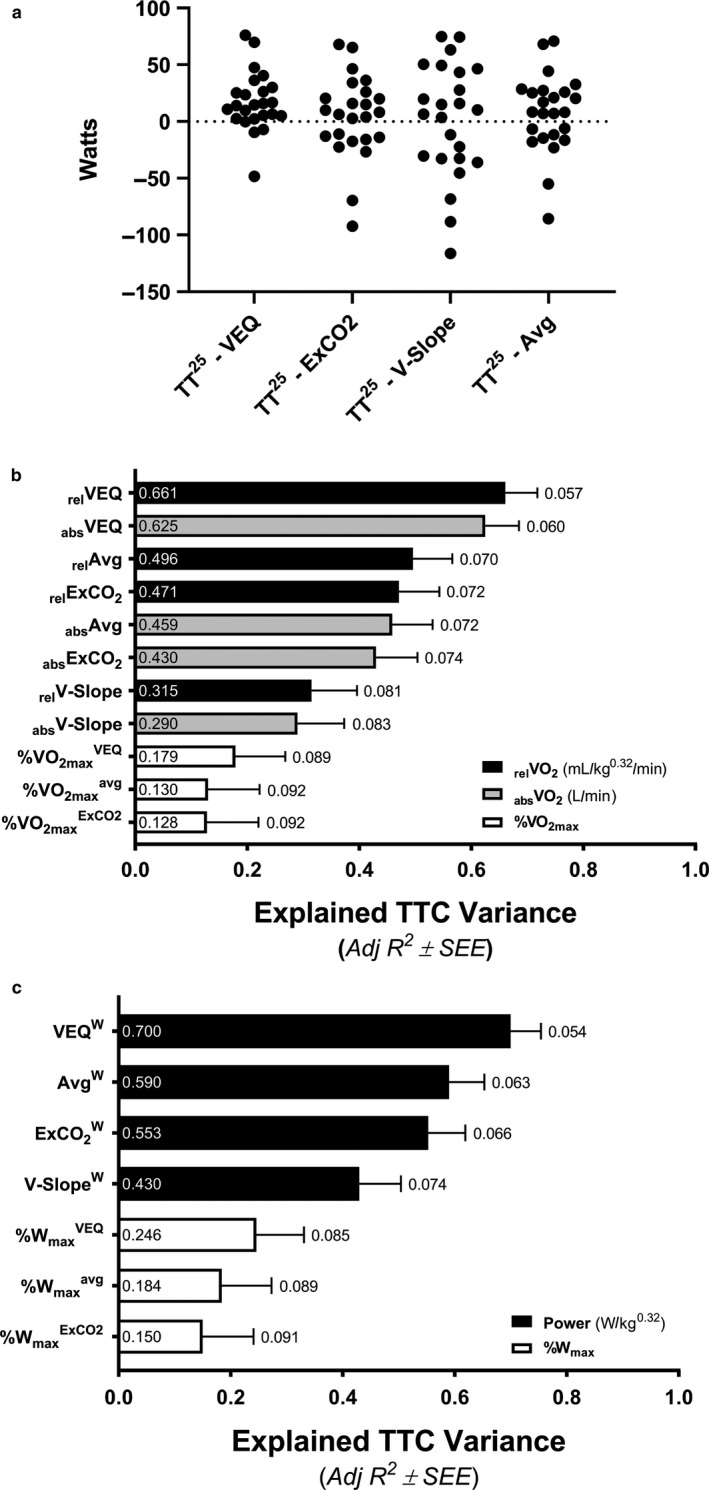
Determination of the best methodology used to identify values at fatigue threshold in relation to human endurance performance. Individual differences between power determined using the ventilatory equivalents (VEQ), excess carbon dioxide (ExCO_2_), modified V‐slope (V‐Slope), ventilatory threshold average (Avg; average of VEQ, ExCO_2_, and V‐Slope values) methods and average 25‐km time trial (TT^25^) power output (a). Linear regression relationships between TT^25^ time‐to‐completion (TTC) and traditional variables representing rates of oxygen consumption (VO_2_; b) or power (c) at fatigue thresholds identified through different methods. The labels within bars reports individual Adj R^2^ values representing explained variance and the labels outside of the error bars represents the standard error of the estimate (SEE) as a percent of the averaged group TTC (2,680.9 s)

All fatigue threshold variables significantly related to TTC are shown in Figure 2b (VO_2_) & 2C (power). The VEQ method in relation to TTC was identified as superior to all other methods used to determine all respective fatigue threshold values. Accordingly, fatigue threshold values determined by the VEQ method (_abs_VO_2_, _rel_VO_2_, %VO_2max_, W, and %W_max_) were included in subsequent grouped forward regression analyses.

#### 3.2.3 Determination of the best indices of cycling efficiency in relation to human endurance performance

At the end of exercise test 1, VO_2max_ and corresponding W_max_ were determined as the highest stable (slope = 0.00005 ± 0.00811) averaged over the longest (51 ± 23 s) possible range. Secondary VO_2max_^1^ confirmation criteria included an RER > 1.10 (1.16 ± 0.05) and HR_max_ within 20 bpm of age‐predicted (Tanaka et al., 2001) HR_max_ (175 ± 11 vs. 182 ± 8 bpm respectively). Maximal rates of VO_2_ were never stable enough at the end of exercise test 2 to utilize least squares linear regression and identify VO_2max_^2^, therefore the only measure of VO_2max_ assessed was derived from exercise test 1.

There was a main effect for method used to determine _abs_VO_2_ (*F* = 6.76, *p* = .0143), _rel_VO_2_ (*F* = 7.375, *p* = .0106), and power (*F* = 48.84, <.0001) at maximal effort; _abs_VO_2peak_^1^ was higher than both _abs_VO_2max_^1^ (*p* = .0002) and _abs_VO_2peak_^2^ (*p* = .0207) with no difference between _abs_VO_2max_^1^ and _rel_VO_2peak_^2^ (*p* = .1753; Figure 1a). Similarly, _rel_VO_2peak_^1^ was higher than both _rel_VO_2max_^1^ (*p* = .0003) and _rel_VO_2peak_^2^ (*p* = .0153) with no difference between _rel_VO_2max_^1^ and _rel_VO_2peak_^2^ (*p* = .1456; Figure 1b). Regarding power at maximal effort, PPO^1^ was higher than W_max_^1^ and W_peak_^1^ (both, *p* < .0001) with no difference between W_max_^1^ and W_peak_^1^ (*p* > .9999; Figure 1c).

While mean differences across methods were significant, the relationship between TTC and measures used to determine _abs_VO_2_ ( *F* = .04402, DFn = 2, DFd = 66, *p* = .9570), _rel_VO_2_ (*F* = .05587, DFn = 2, DFd = 66, *p* = .9457), and power (*F* = .02738, DFn = 2, DFd = 66, *p* = .9730) at maximal efforts were not different (Figure 1d–f respectively). Therefore, corresponding data were averaged and used for subsequent regression analyses. Average measures of VO_2_ and power collected at maximal efforts are referenced as _abs_VO_2max_, _rel_VO_2max_, and W_max_, respectively, throughout the rest of the manuscript.

Linear regression analyses identified _abs_VO_2peak_ (l·min^−1^), _rel_VO_2max_ (ml·kg^−0.32^·min^−1^), and W_max_ (W·kg^−0.32^) as significant in relation to TTC (Figure 1g). Therefore, all three variables were included in subsequent grouped forward regression analyses.

### 3.3 Regression modeling to explain cycling endurance performance

#### 3.3.1 Best traditional grouped variable forward regression models in relation to TTC

Traditional variable grouped forward regression analyses limited initial values included in the model as per suggested correlational collinearity (*R* ^2^ ≥ .80; Figure 4). Corresponding measures of _abs_VO_2_, _rel_VO_2_, and W_max_ were grouped separately for forward regression modeling due to suggested collinearity and to minimize the possibility of overfitting. The best model of TT^25^ performance to include traditional variables reflecting measures collected at maximal effort, fatigue threshold, and exercise efficiency, as suggested by the literature over the past two decades, explained 76.2% of TTC variance (Adj *R* ^2^ = .762, *F* = 25.6, SEE = 128.7, *p* < .001). This model consisted of _abs_VO_2max_ (*p* < .001), %VO_2max_^VEQ^ (*p* = .001), and CE^50^ (*p* = .070) (Figure 5).

(1) $$\text{TTC}=5623.5-\left(339.3\times_{\text{abs}}{\text{VO}}_{2\max}\right)-\left(1271.4\times \%{\text{VO}}_{2\max}^{\text{VEQ}}\right)-\left(17.7\times {\text{CE}}^{50}\right)$$

**Figure 3:**
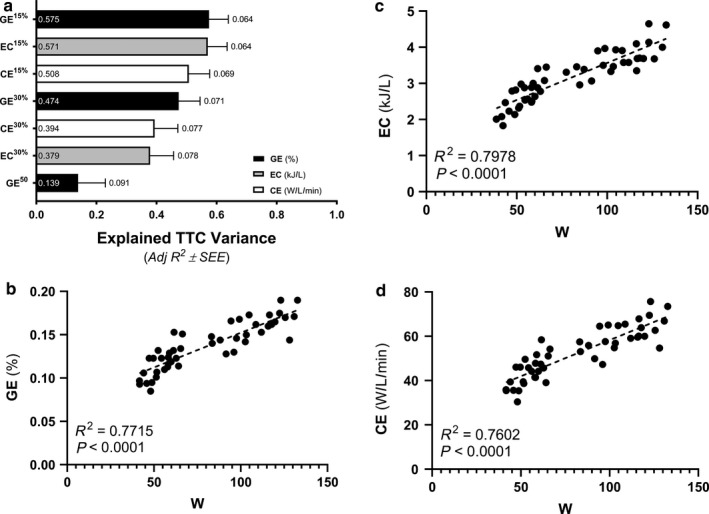
Determination of the best indices of cycling efficiency in relation to human endurance performance. Linear regression relationships between 25‐km time‐to‐completion (TTC) and traditional variables representing gross efficiency (GE), exercise economy (EC), and cycling economy (CE) at 50 W (^50^), 15%, or 30% of power output at the point of exhaustion for exercise test 1 (a). The labels within bars reports individual Adj *R* ^2^ values representing explained variance and the labels outside of the error bars represents the standard error of the estimate (SEE) as a percent of the averaged group TTC (2,680.9 s). Indices of exercise efficiency, including GE (b), EC (c), and CE (d), all improved in relation to higher cycling workloads during exercise test 2

**Figure 4:**
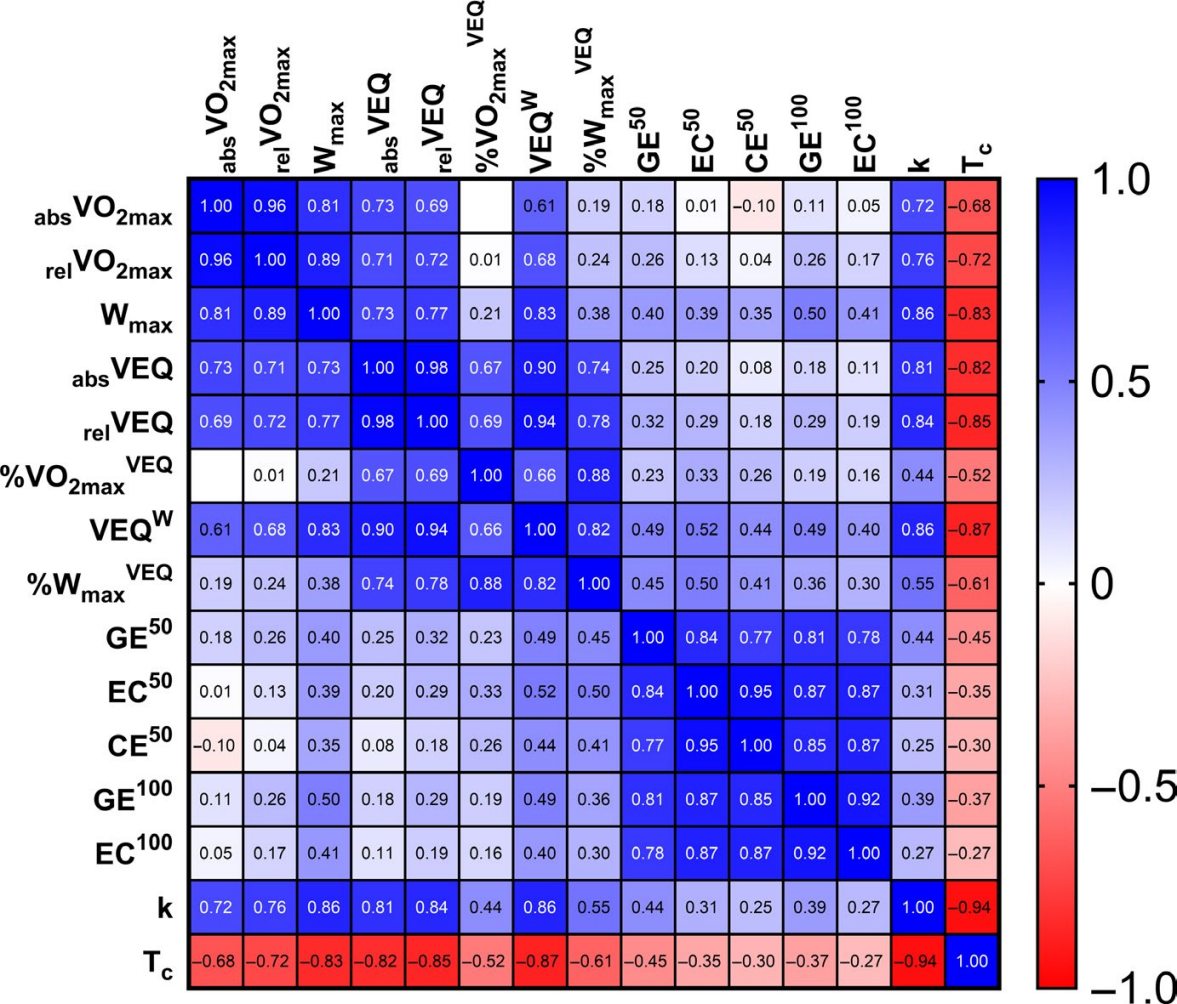
Shown above is a correlation matrix of all this study's best traditional variables typically used to describe human endurance performance along with indices of maximal rates of skeletal muscle respiration. Variables include absolute (_abs_; L·min^−1^) rates of whole‐body oxygen consumption (VO_2_), relative (_rel_; ml·min^−1^·kg^−0.32^) VO_2_, power (W·kg^−0.32^), gross efficiency (GE), exercise economy (EC), cycling economy (CE), rate constant for the recovery of skeletal muscle oxygen consumption (k) or the reciprocal time constant for the recovery of skeletal muscle oxygen consumption (T_c_). Traditional measures of VO_2_ or power collected at maximal efforts were averaged (_max_). All fatigue thresholds were determined using the ventilatory equivalents (VEQ) method. Indices of exercise efficiency were determined during cadence‐controlled cycling (80 rpm) at either 50 or 100 W

**Figure 5:**
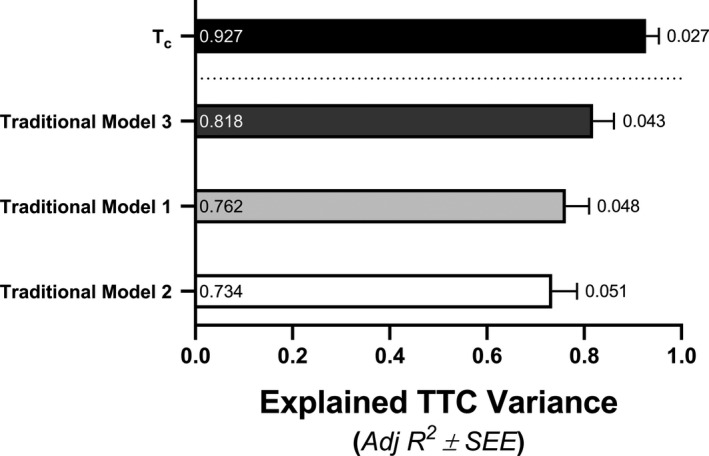
Linear regression models determined via forward regression analyses in relation to 25‐km time‐to‐completion (TTC). The labels within bars reports individual Adj *R* ^2^ values representing explained variance and the labels outside of the error bars represents the standard error of the estimate (SEE) as a percent of the averaged group TTC (2,680.9 s). The time constant for the recovery of skeletal muscle oxygen consumption (T_c_); Traditional regression models 1, 2, and 3 are defined in the Results section under the section entitled, Regression modeling to explain cycling endurance performance

The next best traditional model included _rel_VO_2max_ (*p* = .036), _abs_VEQ (*p* = .022), and CE^50^ (*p* = .022), which explained 73.4% of cycling endurance performance (Adj *R* ^2^ = .734, *F* = 22.1, SEE = 136.1, *p* < .001).

(2) $$\text{TTC}=4789.6-\left(0.655\times_{\text{rel}}{\text{VO}}_{\text{2max}}\right)-\left(290.9\times_{\text{abs}}\text{VEQ}\right)-\left(16.2\times {\text{CE}}^{50}\right)$$

The last traditional model, explaining 81.8% of TTC variance, included W_max_ (*p* < .001), %VO_2max_^VEQ^ (*p* = .003), and GE^100^ (*p* = .204). However, GE^100^ did not significantly contribute to the model with the combination of W_max_ (*p* < .001) and %VO_2max_^VEQ^ (*p* = .005) primarily predicting TTC (Adj *R* ^2^ = .812, *F* = 50.6, SEE = 114.5, *p* < .001).

(3) $$\text{TTC}=4791.1-\left(17.0\times W_{\max}\right)-\left(958.2\times \%{\text{VO}}_{2\max}^{\text{VEQ}}\right)$$

#### 3.3.2 Best grouped variable forward regression models in relation to TTC including measures of maximal skeletal muscle respiration

**Figure 6:**
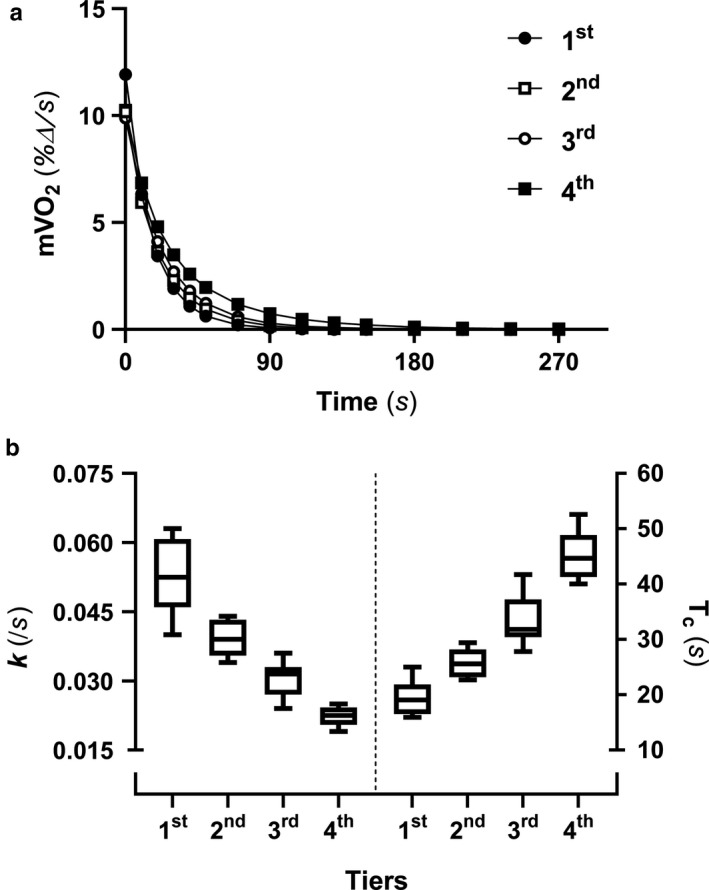
Measures of skeletal muscle respiration. The percent change in rates of skeletal muscle oxygen consumption (mVO_2_) over time (a) was used to determine the rate (k) and time constants (T_c_) for the recovery of mVO_2_ (b). Values are separated into quartiles representing the fastest (1st) to the slowest (4th) 25‐km times to completion, with an *n* = 6 in each quartile (total *n* = 24). The whiskers represent 95% confidence intervals

Values showing rates of mVO_2_ recovery, k, and T_c_ across performance quartiles are shown in Figure 6. Forward regression analyses were assessed with k or T_c_ along with all other top traditional variables excluding those identified as collinear with the NIRS‐derived measures of maximal skeletal muscle respiration (Figure 4). The most complete model regarding TT^25^ performance included only T_c_, which, alone, explained 92.7% of TTC variance (Adj *R* ^2^ = .927, *F* = 294.2, SEE = 71.2, *p* < .001).

(4) $$\text{TTC}=1930.95+\left(24.25\times T_{c}\right)$$

Independent variables used for regression modeling were normally distributed (Shapiro‐Wilk test) and all regression analyses (Figure 5) were reported to have sufficient power (all, 1.000) to perform tests with α = .05 (SigmaPlot, Systat).

## 4 DISCUSSION

The present study scrutinized biological predictors of human cycling endurance performance by examining the explained variance of a 25‐km time‐trial (TT^25^) time‐to‐completion (TTC) across a well‐defined group of trained adult males (*n* = 24). Our most important and novel finding is that the time constant for the rate of skeletal muscle oxygen consumption (mVO_2_) recovery, T_c_, alone, provided the most complete model of cycling endurance performance by explaining 92.7% of TTC variance across participants. The best model of endurance performance using traditional variables were not as robust. The maximal rate of whole‐body oxygen (O_2_) consumption (l·min^−1^; _abs_VO_2max_), %VO_2max_ determined by the ventilatory equivalents method (%VO_2max_^VEQ^), and cycling economy assessed at 50 W (CE^50^) collectively explained 76.2% of endurance performance variance. A more complete traditional model that included maximal power output (W_max_) and %VO_2max_^VEQ^, which could not be strengthened by any index of exercise efficiency, only explained 81.2% of TTC variance. This is the first study to verify that traditional variables including measures obtained at maximal effort, a valid fatigue threshold value, and an index of exercise efficiency do collectively explain a majority of cycling endurance performance (i.e. >75% variance). Additionally, this is the first study to demonstrate the relationship between NIRS‐derived maximal rates of skeletal muscle respiration and cycling endurance performance. These findings expand upon and improve the clarity of how human biology directs cycling endurance performance by highlighting the importance of skeletal muscle respiratory potential over classically held traditional variables in relation to the limits of human performance.

The positive relationship between exercise training, mitochondrial adaption in skeletal muscle, and exercise performance is well‐documented. Skeletal muscle mitochondrial protein content (Granata, Jamnick, & Bishop, 2018; Holloszy, 1967; Robinson et al., 2017), volume density (Granata et al., 2018; Hamaoka et al., 1996; Meinild Lundby et al., 2018), and maximal rates of respiration (Granata et al., 2018; Jacobs et al., 2013; Pesta et al., 2011) increase with endurance‐type exercise training. These adaptations help facilitate training‐induced improvements in endurance capacity (Jacobs et al., 2013; Lundby & Jacobs, 2016). Skeletal muscle respiratory potential contributes to the biological mechanisms underlying both VO_2max_, as detailed by the Fick equation, and one's fatigue threshold. Skeletal muscle specific capillary density (Hoppeler et al., 1985; Zumstein et al., 1983), mitochondrial volume density (Hoppeler et al., 1985; Hoppeler, Lüthi, Claassen, Weibel, & Howald, 1973; Lundby & Jacobs, 2016), mitochondrial protein content (Blomstrand, Rådegran, & Saltin, 1997; Jacobs & Lundby, 2011), and measures of maximal cellular respiration (Ivy, Costill, & Maxwell, 1980; Jacobs & Lundby, 2013; Jacobs et al., 2012; Pesta et al., 2011) all correlate with VO_2max_. High‐intensity interval training can increase maximal rates of skeletal muscle respiration, VO_2peak_, and endurance performance independent of changes in maximal cardiac output, blood volumes, and oxygen carrying capacity of the blood (Jacobs et al., 2013). The strong relationships (*R* ^2^ > .80) between fatigue thresholds and maximal rates of skeletal muscle respiration (Ivy et al., 1980), which were confirmed in the current study (Figure 4), emphasize the primary role of skeletal muscle bioenergetic oxidative potential in determining one's fatigue threshold during exercise (Holloszy & Coyle, 1984; Poole et al., 2016; Vanhatalo et al., 2016). Maximal rates of skeletal muscle respiration obtained via high‐resolution respirometry have also been identified as the single best predictor of cycling endurance performance in highly‐trained‐to‐elite level athletes (Jacobs et al., 2013). The biological relationship between VO_2max_, fatigue thresholds, and skeletal muscle oxidative potential most likely explain why maximal rates of skeletal muscle respiration more completely describe endurance performance (Figure 5) when compared to other traditional variables commonly referenced in relation to the limits of human endurance potential across decades of human performance research (Coyle, 1995; Gabriel & Zierath, 2017; Joyner & Coyle, 2008).

Alternatively, abrogating training‐induced improvements in skeletal muscle oxidative potential reciprocally blunts improvements in cardiorespiratory fitness, endurance capacity, and, sometimes, measures of whole‐body health. Supplementation of exogenous vitamin C with exercise training can blunt mitochondrial biogenesis in skeletal muscle (Bruns et al., 2018; Gomez‐Cabrera et al., 2008) and minimize improvements in VO_2max_ and/or endurance performance in both humans and rats (Gomez‐Cabrera et al., 2008). Diminished skeletal muscle mitochondrial protein is reported to parallel increases in heart rate, ventilation, respiratory exchange ratio, and blood lactate concentrations at the same absolute submaximal exercise intensity following 84 days of detraining (Coyle, Martin, Bloomfield, Lowry, & Holloszy, 1985). Higher VO_2max_ and lactate threshold values have also been attributed to approximately 50% higher measures of skeletal muscle capillarization and mitochondrial protein in detrained endurance athletes compared to sedentary controls with no history of exercise training (Coyle et al., 1985, 1984). More recently, metformin was shown to abolish exercise‐induced increases in skeletal muscle respiration and attenuate concomitant improvements to VO_2max_ and whole‐body insulin sensitivity using a randomized double‐blind placebo‐controlled design. Training‐induced changes in skeletal muscle respiration also correlated with changes in whole‐body insulin sensitivity (Konopka et al., 2019). The importance of skeletal muscle oxidative potential most likely extends past the relationship with endurance performance, as the whole‐body health benefits of exercise training track with improvements in exercise performance (Gabriel & Zierath, 2017). However, until recently, our ability to assess dynamic measures of skeletal muscle mitochondrial function relied primarily on access to an MRI scanner or the more invasive collection of a skeletal muscle via tissue biopsy.

NIRS‐derived measures of skeletal muscle oxygenation allow for the direct noninvasive assessment of maximal rates of skeletal muscle respiration (Hamaoka et al., 1996; Ryan et al., 2014; Ryan, Southern, Reynolds, et al., 2013), with a precision capable of differentiating skeletal muscle oxidative potential across human participants with differing levels of fitness (Brizendine, Ryan, Larson, & McCully, 2013; Lagerwaard et al., 2019) and within‐subject skeletal muscle respiratory changes contrasting trained and nontrained arms (Ryan, Southern, Brizendine, & McCully, 2013). NIRS‐derived measures of maximal skeletal muscle respiration have been validated against high‐resolution respirometry‐derived measures of maximal ADP‐stimulated state 3 respiration (Ryan et al., 2014) using an in situ mitochondrial model with permeabilized muscle sample preparations that leave the mitochondrial reticular network intact (Kuznetsov et al., 2008) and in vivo PCr recovery kinetics via ^31^phosphorus‐magnetic resonance spectroscopy (Ryan, Brizendine, et al., 2013). A benefit of this NIRS methodology is that it allows for maximal rates of skeletal muscle respiration to be assessed without the collection of a skeletal muscle biopsy and away from a costly MRI scanner. This technique, originally tested in 1996 (Hamaoka et al., 1996) and later modified to control for blood volume changes with ischemic limb cuffing in 2012 (Ryan et al., 2012), could greatly improve practical applications of skeletal muscle mitochondrial assessments. Indeed, this study confirms previous findings (Jacobs et al., 2011), which were obtained through skeletal muscle biopsy sampling and high‐resolution respirometry, showing that measures reflecting integrative and dynamic rates of maximal skeletal muscle respiration can strongly predict human endurance performance. More research is needed to fully examine the benefit of this procedure as well as general NIRS‐derived measures of skeletal muscle oxygenation during exercise to possibly improve the precision with which individualized training can be monitored. NIRS‐derived measures of skeletal muscle oxygenation during exercise improved regression modeling to explain 26.15‐km (Jacobs et al., 2011) and 15‐km (van der Zwaard et al., 2018) time trial performances. These data provide the proof‐of‐concept necessary to further explore the use of skeletal muscle oxygenation assessment similar to, or along with, the use of heart rate during exercise to provide a real‐time assessment of biological performance with respect to external measures of performance (i.e. power output, running velocity, etc.).

This study is the first study to completely validate the long‐held premise that a combination of traditional variables derived from three separate physiologic tiers representing one's cardiorespiratory fitness, fatigue threshold, and bioenergetic efficiency during exercise, respectively, account for the majority of cycling endurance performance (i.e. >75%). The combination of _abs_VO_2max_, %VO_2max_^VEQ^, and CE^50^ accounted for 76.2% of endurance performance variance. Many studies have identified one or two necessary components of the traditional variable postulate as predictive of endurance performance (Amann et al., 2006, 2004; Coyle, Coggan, Hopper, & Walters, 1988; Coyle et al., 1991; Lamberts et al., 2012; van der Zwaard et al., 2018). More recently a group from The Netherlands confirmed that VO_2max_, fatigue threshold VO_2_s derived using both LT and VT methods, and GE all independently correlated with 15‐km time trial performance (van der Zwaard et al., 2018). Yet, there was no mention of a collective 3‐tier model including variables relating to maximal effort, fatigue threshold, and exercise efficiency in relation to cycling performance. They did report that performance VO_2_, defined as the average rate of O_2_ consumption over a 15‐km time trial test, explained 88% of the variance for 15‐km time trial performance (van der Zwaard et al., 2018). A product of VO_2max_ and LT (4 mmol/·L), VO_2(LT2)_, was reported to explain up to 93% of the variance of performance VO_2_ across cyclists, which should translate to ~80% of 15‐km time trial performance variance. The combination of VO_2max_ and VO_2(LT2)_ explaining ~80% of 15‐km time trial performance variance (van der Zwaard et al., 2018) is similar to our two‐variable model of performance that included W_max_ and %VO_2max_^VEQ^ and explained 81.2% of TTC variance. Measures of cycling power at maximal efforts, which also share a stronger relationship to maximal rates of skeletal muscle respiration (Figure 4), have been reported as more complete than respective measures of VO_2_ (Hawley & Noakes, 1992; Lamberts et al., 2012). Measures of power at maximal effort, however, provide minimal mechanistic insight regarding the biological parameters that are primarily responsible for setting the limits on human endurance performance.

Of note, VO_2max_ more completely explained 15‐km time trial performance (*R* ^2^ = .79) across a group of cyclists who competed at the national, international, or Olympic levels (van der Zwaard et al., 2018) than in the current study that included recreational‐to‐highly‐trained participants (Figure 1g). Together these findings highlight the inconsistencies and potential methodological pitfalls when relying on VO_2max_ to describe endurance performance potential, as is too common across exercise science research. An example of such research includes inappropriately controlled studies testing the live‐high train‐low model to improve sea level performance with VO_2max_ as the only measure of performance (Jacobs, 2013).

### 4.1 Study limitations

**Figure 7:**
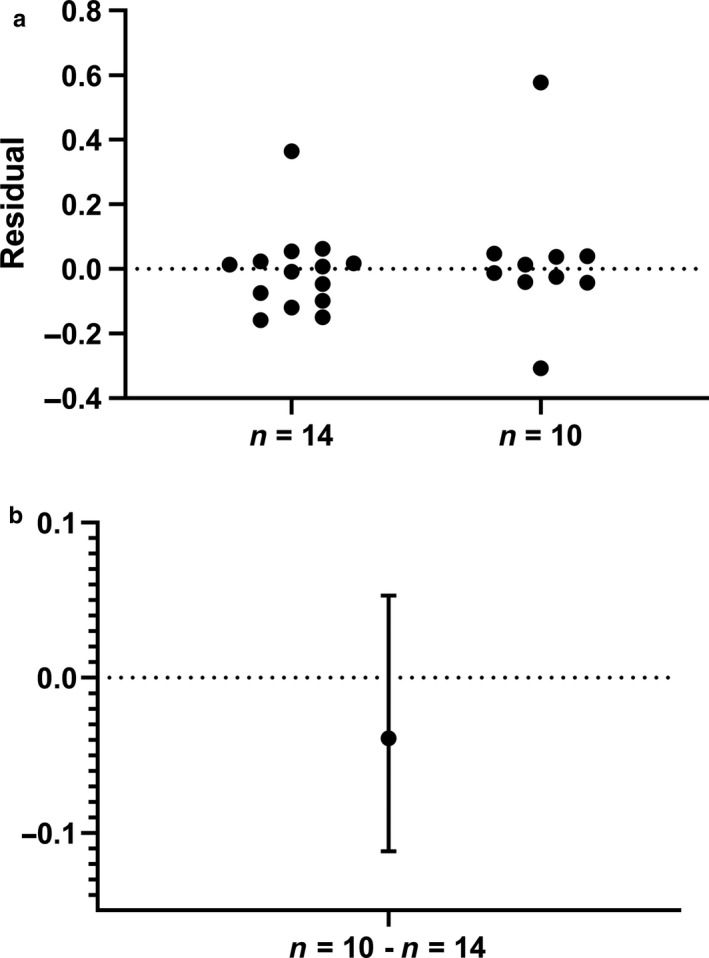
Comparison of the power ratio, defined as the average 25‐km time trial power to individual fatigue threshold power identified using the ventilatory equivalents method, in the fastest (*n* = 14) versus the slowest (*n* = 10) study participants. Residuals from the Mann–Whitney test are plotted in a whereas the median difference and 95% confidence interval is shown in b

The exercise tests conducted in this study were all obtained on the same day within the span of ~4 hr. No familiarization trials were conducted. We do not believe this decision influenced our primary outcome variables, as our data corresponds with previously published literature. For example, Lamberts et al. (2012) examined predictors of 40‐km time trial performance across a group of highly trained competitive male cyclists (*n* = 45) in Cape Town, South Africa (~40 m elevation). Their study participants completed familiarization tests 72 hr prior to an incremental ramp test to collect variables at maximal effort and a 40‐km time trial (Lamberts et al., 2012). While we did not conduct familiarization tests, a subsample (*n* = 14) of participants from our entire sample group (*n* = 24), consisting of our top cyclists, shared near identical individual characteristics (age, height, weight, VO_2max_, PPO, etc.) and measures of performance (average time trial speed and power output) across independent studies (Table 4). Moreover, there was no difference between the ratio of power averaged throughout the TT^25^ to W^VEQ^ in those 14 study participants when compared to the additional 10 (Figure 7), which collectively make up the entire sample group for this study. The similarities across independent studies suggest that the lack of familiarization tests in the current study did not greatly influence the results.

**Table 4:** This table reports study participant characteristics and indices of cycling endurance performance from a subset (*n* = 14) of top performers in the current study collected at ~1,950 m alongside published values derived from highly‐trained cyclists (Lamberts et al., 2012) collected at ~40 m

Sample Group	Current Study	Lamberts et al. (2012)
Sample size	*n* = 14	*n* = 45
Age (years)	35 ± 12	32 ± 6
Height (cm)	180.6 ± 4.8	180.7 ± 7.4
Body Mass (kg)	76.3 ± 6.7	76.8 ± 7.5
Power at maximal effort (W)	380 ± 30	381 ± 42
Maximal power‐to‐weight ratio (W·kg^−1^)	5.1 ± 0.5	5.0 ± 0.5
Maximal power‐to‐weight ratio (W·kg^−0.32^)	95.1 ± 7.7	95.1 ± 9.4
VO_2max_ (l·min^−1^)	4.4 ± 0.4	4.4 ± 0.5
VO_2max_ (ml·min^−1^·kg^−1^)	57.3 ± 6.5	57.3 ± 6.2
VO_2max_ (ml·min^−1^·kg^−0.32^)	1,086.8 ± 100.3	1,093.9 ± 121.5
Time Trial Length (km)	25	40
TTC (s)	2,494 ± 121	3,964 ± 181
Speed (km·hr^−1^)	36.1 ± 1.8	36.3 ± 1.7
Average TT power (W)	250 ± 33	254 ± 32
Power‐to‐weight ratio (W·kg^−1^)	3.30 ± 0.57	NR (~3.31 ± 0.42)
Power‐to‐weight ratio (W·kg^−0.32^)	62.6 ± 8.7	NR (~63.3 ± 8.0)

Note

Data reported as mean ± *SD*.

Abbreviations: NR, not reported; TT, time trial; TTC, time‐to‐completion; VO_2max_, maximal rate of whole‐body oxygen consumption.

Growing evidence suggests that critical power, which was not assessed in the current study, may be the most appropriate fatigue threshold method to assess threshold or functional power output in relation to endurance performance (Jones et al., 2019). Critical power has been shown to relate to cycling endurance performance better than various measures of power at VT (Black et al., 2014; Smith, Dangelmaier & Hill 1999) while measures of power at VT have been reported as superior to respective powers at LT (Amann et al., 2006). Given the complexities, assortment of methodologies, subjective interpretations and/or additional burden of obtaining VT and, especially, LT values, we agree, in principal, with the benefit of determining critical power. Consequently, we have implemented a 3‐min all‐out test of critical power (Vanhatalo et al., 2007) in our ongoing research in place of the verification max test utilized in the current study, 110% PPO until volitional fatigue. We cannot, however, rule out that measures of CP may have improved upon some of our ‘traditional’ regression models of performance. This decision was in part attributed to claims suggesting that the value of critical power is limited to high‐intensity exercise efforts lasting under 10 min (Currell & Jeukendrup, 2008) and previous evidence demonstrating the strength of VT values, especially those determined by VEQ, in relation to 40‐km time trial cycling performance (Amann et al., 2006).

Lastly, all our measures of cycling endurance performance were collected in a controlled environment on a stationary cycle ergometer. While we utilized empirically derived data suggested to improve the translation of stationary laboratory cycling tests to real‐world application by allometrically scaling necessary data to account for relationships between body mass, frontal area, and air resistance/drag when cycling outside on flat terrain (Swain, 1994), the results presented here should be reexamined in real‐world conditions to validate these findings.

## 5 SUMMARY

This study is the first to verify and validate the traditional predictive theoretical model of endurance performance based on one's VO_2max_, a valid fatigue threshold, and an index of exercise efficiency to explain the majority (i.e. >75%) of cycling endurance performance. More importantly, however, this is the first study to demonstrate that one noninvasive NIRS‐derived measure reflecting maximal rates of skeletal muscle respiration improves upon the predictive power of the traditional model accounting for 92.7% of endurance performance variance across trained study participants (*n* = 24). When compared to 43 traditional variables long postulated to predict human endurance performance, measures reflecting maximal rates of skeletal muscle respiration arguably stand alone as the best. E pluribus unum; out of many, one.

## CONFLICT OF INTEREST

The authors declare that no potential competing interests exist.

## AUTHOR CONTRIBUTIONS

Conceived and designed the experiments (RAJ), performed the experiments (RAJ, PMB, MRN, SEH, and SR), analyzed the data (RAJ, PMB, MRN and KGL), discussed and interpreted the results (RAJ, PMB, AWS, and KGL), wrote the paper (RAJ and PMB), assisted with paper revisions (RAJ, PMB, MRN, SEH, SR, KGL, and AWS), approved the final version of the paper (PMB, MRN, SEH, SR, KGL, AWS, and RAJ).
